# Multi-model genome-wide association analysis reveals natural allelic variation and candidate loci for kernel phytic acid content in quality protein maize

**DOI:** 10.3389/fgene.2026.1925511

**Published:** 2026-10-12

**Authors:** Sushil Kumar, Sourabh Kumar, Dinesh Kumar Saini, Rainy Rainy, Abhijit Kumar Das, Chikkappa G. Karjagi, Dharampaul Chaudhary, Shailendra Singh Gaurav, Ramesh Kumar

**Affiliations:** 1 Indian Council of Agricultural Research (ICAR)-Indian Institute of Maize Research, Ludhiana, Punjab, India; 2 Department of Genetics and Plant Breeding, Chaudhary Charan Singh University, Meerut, Uttar Pradesh, India; 3 Department of Plant and Soil Science, Texas Tech University, Lubbock, TX, United States; 4 School of Agricultural Biotechnology, Punjab Agricultural University, Ludhiana, Punjab, India; 5 Regional Maize Research and Seed Production Centre, ICAR-Indian Institute of Maize Research, Vishnupur, Bihar, India

**Keywords:** biofortification, candidate genes, genome-wide association study, phytic acid, quality protein maize

## Abstract

Phytic acid (PA) is the major phosphorus-storage compound in maize kernels, but its strong mineral-chelating capacity limits the bioavailability of essential nutrients in humans and monogastric animals. Although classical low-phytic-acid mutants have demonstrated the feasibility of phytate reduction, their use in breeding has been constrained by pleiotropic effects on seed viability, germination, and agronomic performance. Therefore, identifying natural allelic variation for kernel PA content in elite nutritional backgrounds such as Quality Protein Maize (QPM) is important for biofortification breeding. To our knowledge, no genome-wide association study has yet been reported for kernel PA content in maize. Here, we evaluated 249 QPM inbred lines across two environments and conducted genome-wide association analysis using sequencing-derived SNP markers. Kernel PA content showed substantial phenotypic variation, highly significant genotypic effects, and high broad-sense heritability in both environments, indicating strong genetic control. Of 11,525,180 variants initially called, 115,416 chromosomal biallelic SNPs passed initial filtering, and 26,074 high-quality SNPs were retained for population structure, linkage disequilibrium, and association analyses. Multi-model GWAS identified five high-confidence marker-trait associations on chromosomes 1, 3, 4, and 7. The strongest locus, chr3:236714544, was consistently detected across environments and analytical models and explained up to 17.7% of phenotypic variation. Allelic effect analysis confirmed significant differences among genotype classes at all five loci, with the clearest differentiation observed at chr3:236714544. LD-based haplotype analysis identified a three-SNP chromosome four haplotype block associated with kernel PA variation, with Hap2/GAC showing lower PA content. Integrated candidate-gene analysis prioritized seven putative candidate genes involved in transcriptional regulation, phosphate transport, phosphorylation-dependent signaling, seed development, and lipid/protein storage-related processes. *Zm00001eb163880* emerged as the strongest candidate gene at the major chr3:236714544 locus, supported by a predicted missense variant and strong seed-related expression. Together, these results identify robust genomic loci, favorable allelic variation, and prioritized candidate genes for kernel PA content, providing a foundation for marker-assisted selection and future validation toward developing QPM maize with reduced anti-nutritional burden and improved mineral bioavailability.

## Introduction

Maize (*Zea mays* L.) is one of the world’s most important staple crops, supporting food, feed, and industrial systems across diverse production environments. It plays a central role in global food security, particularly in low-income regions of Africa, Latin America, and Asia, where maize-based diets contribute substantially to daily caloric intake (Shiferaw et al., 2011). Maize grain is also a major component of livestock and poultry feed, and increasing demand for animal-source foods has further expanded its importance in feed and bio-industrial sectors (Shiferaw et al., 2011; Shahzad et al., 2014; Erenstein et al., 2022). However, despite its high productivity and caloric value, normal maize is nutritionally constrained by poor protein quality and limited micronutrient bioavailability. The major endosperm storage proteins, zeins, are inherently deficient in the essential amino acids (*viz.*, lysine and tryptophan), thereby reducing the biological value of conventional maize protein (Mertz et al., 1964; Prasanna et al., 2001). In addition, cereal-based diets often contain anti-nutritional compounds that restrict mineral absorption, thereby contributing to micronutrient malnutrition or “hidden hunger” in populations dependent on staple grains (Shahzad et al., 2014).

One of the earliest genetic breakthroughs in maize nutritional improvement was the discovery of the *opaque2* mutant, which altered endosperm protein composition and increased kernel lysine content (Mertz et al., 1964). Subsequent breeding efforts incorporated modifier genes into the *opaque2* background, leading to the development of Quality Protein Maize (QPM), which combines enhanced lysine and tryptophan content with improved kernel hardness and agronomic acceptability (Vasal, 2000; Prasanna et al., 2001; Maqbool et al., 2021). Current maize biofortification programs increasingly aim to combine multiple nutritional traits, including improved protein quality, provitamin A enrichment, and enhanced mineral density (Harjes et al., 2008; Shahzad et al., 2014; Yan et al., 2023; Sethi et al., 2023; Gedil et al., 2024; Jasrotia et al., 2026). However, one major biochemical barrier remains: phytic acid (PA), or myo-inositol-1,2,3,4,5,6-hexakisphosphate, in maize kernels. Even when grain mineral concentration is improved, high PA levels can limit mineral bioavailability and reduce the nutritional impact of biofortification (Raboy, 2003; Bohn et al., 2008).

Phytic acid is the principal storage form of phosphorus in cereal grains and commonly accounts for a major proportion of seed total phosphorus (Raboy, 2003; Shi et al., 2007). In maize kernels, phytate accumulates mainly during grain filling and is stored largely in embryo and aleurone-associated tissues as mixed salts with potassium, magnesium, and other cations (Raboy, 2003; Shi et al., 2007). From the plant perspective, PA functions as an important reservoir of phosphorus and minerals required for germination and early seedling establishment. Nutritionally, however, PA is a strong anti-nutritional factor because its highly negative phosphate groups chelate mineral cations such as Fe^2+^, Zn^2+^, Ca^2+^, and Mg^2+^, forming insoluble phytate-mineral complexes that are poorly digested by humans and monogastric animals (Gupta et al., 2015; Brouns, 2021). Consequently, high phytate content in cereal-based diets reduces the bioavailability of iron, zinc, and other essential minerals. In livestock production, undigested phytate phosphorus is excreted in manure, contributing to phosphorus loading and eutrophication in aquatic systems (Raboy et al., 2000; Bohn et al., 2008). Reducing kernel PA is therefore important for improving both nutritional quality and environmental sustainability.

Classical genetic studies have demonstrated that low-PA phenotypes are biologically attainable. In maize, the *lpa1-1* and *lpa2-1* mutants substantially reduce seed phytate phosphorus while increasing inorganic phosphorus, often without major changes in total phosphorus concentration (Raboy et al., 2000). Similar low-PA mutants have also been reported in other cereal and legume crops, confirming that phytate accumulation can be genetically modified (Cominelli et al., 2020). However, the breeding application of induced *lpa* mutants has often been constrained by undesirable pleiotropic effects, including reduced seed viability, poor germination, reduced seedling vigor, altered seed weight, and yield penalties (Raboy et al., 2000; Cominelli et al., 2020). These trade-offs likely reflect the physiological role of PA in seed development, phosphorus storage, and early seedling growth. Thus, although *lpa* mutants validate the value of phytate reduction, their limitations highlight the need to identify naturally occurring alleles or regulatory variants that can reduce kernel PA without severely disrupting seed performance.

Kernel PA content is a quantitative trait controlled by multiple loci and influenced by genetic background and environment. In maize, QTL mapping has identified genomic regions associated with grain PA concentration under contrasting nitrogen conditions, suggesting that natural genetic variation for this trait can be dissected and used in breeding (Huang, 2013). Environmental effects on PA accumulation and the identification of stable low-phytic-acid maize genotypes have also been reported, further emphasizing the importance of multi-environment evaluation (Pramitha et al., 2020). In other crops, genome-wide association studies have revealed marker-trait associations and candidate genes for PA and related anti-nutritional traits, including grain PA in wheat (Wen et al., 2022) and nutritional/anti-nutritional seed traits in mungbean (Sinha et al., 2023). These studies demonstrate that natural allelic variation for PA exists across crop species and can be exploited through genomics-assisted breeding. However, the genetic basis of PA variation remains insufficiently characterized in nutritionally improved maize backgrounds such as QPM.

Genome-wide association study (GWAS) provides a high-resolution strategy for dissecting complex quantitative traits in diverse germplasm panels. By combining dense genome-wide SNP markers with correction for population structure and kinship, GWAS can identify marker-trait associations that are relevant to breeding populations (Xiao et al., 2017; Saini et al., 2022). Multi-locus and iterative GWAS models can further improve detection power for small-to moderate-effect loci while reducing false positives (Wang et al., 2016; Liu et al., 2016; Huang et al., 2019; Zhang et al., 2020; Lü et al., 2025; Li et al., 2026). For nutritional traits, integrating GWAS with allelic effect analysis, linkage disequilibrium-based haplotype analysis, candidate gene annotation, variant-effect prediction, and expression profiling can strengthen biological interpretation and improve prioritization of candidate genes (Wen et al., 2022; Sinha et al., 2023; Haley et al., 2026). Such integrative approaches are particularly useful for traits such as kernel PA content, where both metabolic function and seed-development context are important.

Despite progress in maize biofortification, GWAS-based dissection of natural variation for kernel PA content has not yet been reported in maize. To our knowledge, this is the first GWAS-based study of kernel PA content in maize, with specific focus on QPM germplasm. Identifying favorable alleles and haplotypes for reduced PA in QPM would provide an important route for combining improved protein quality with enhanced mineral bioavailability. To address this gap, the present study evaluated kernel PA content in a panel of 249 elite QPM inbred lines across two environments and performed multi-model GWAS using sequencing-derived SNP markers. The specific objectives were to: (i) assess phenotypic variation and genetic variability for kernel PA content in QPM germplasm; (ii) identify robust marker-trait associations using complementary GWAS models; (iii) evaluate allelic and haplotype effects of significant loci; and (iv) prioritize candidate genes through functional annotation, variant-effect prediction, and *in silico* expression profiling. The findings are expected to provide genomic resources for marker-assisted selection and precision biofortification of QPM maize with reduced PA content.

## Materials and methods

### Plant material and field experiments

A diversity panel comprising 249 QPM inbred lines was evaluated for kernel PA content across two distinct agro-climatic locations in India. The panel comprised QPM breeding materials maintained or developed at ICAR-IIMR, including lines derived from CIMMYT germplasm; genotype-wise source and pedigree information is provided in Supplementary Table S1. The first trial was conducted at the Agricultural Research Station, Peddapuram, Kakinada, Andhra Pradesh (17.0756° N, 82.1360° E), during the Rabi 2021 season. The second trial was conducted at the ICAR-Indian Institute of Maize Research (IIMR), Ladhowal, Ludhiana, Punjab (30.9020° N, 75.8083° E), during the Spring 2022 season. These environments were designated as AP2021 (E1) and PUN2022 (E2), respectively. Data from E1 and E2 were also combined for across-environment analysis and are referred to as the pooled dataset (E3). The field experiments were laid out in an alpha-lattice design with two replications. Each genotype was planted in a single 3-m row, with 15 plants per row, 65 cm row-to-row spacing, and 20 cm plant-to-plant spacing. Standard agronomic practices and crop protection measures were followed to minimize experimental variation due to biotic and abiotic stresses. To produce selfed seed and prevent pollen contamination, controlled self-pollination was performed by bagging tassels and ears before anthesis. At physiological maturity, selfed cobs were harvested after the appearance of the black layer at the base of the kernel, dried, and processed for PA estimation.

### Kernel phytic acid estimation

Phytic acid content in maize kernels was estimated using the modified Wade reagent colorimetric method (Lorenz et al., 2007), with minor modifications. The assay is based on the decolorization of the pink ferric chloride-sulfosalicylic acid complex by phytate, and the reduction in color intensity was measured spectrophotometrically at 490 nm. For each genotype, two biological replicates were analyzed. Approximately 15–20 mature kernels were ground into a fine powder, and 0.1 g of maize flour was transferred to a 2 mL microcentrifuge tube. Phytic acid was extracted by adding 2 mL of 0.65 M HCl, followed by overnight shaking at room temperature. The 0.65 M HCl solution was prepared by adding 5.4 mL of 36% HCl and making the final volume to 100 mL with distilled water. The samples were centrifuged at 3,000 rpm for 20 min at room temperature, and the clear supernatant was used for colorimetric estimation. For each sample, 0.3 mL of supernatant was mixed with 2 mL of freshly prepared working Wade reagent. The Wade reagent stock was prepared by dissolving 2.5 g of 5′-sulfosalicylic acid and 0.25 g of ferric chloride hexahydrate in 150 mL distilled water, adjusting the pH to 3.05 with NaOH, and making the final volume to 200 mL. The working Wade reagent was prepared by diluting the stock reagent with distilled water in a 1:4 ratio before use. The reaction mixture was incubated in the dark for 20 min, and absorbance was recorded at 490 nm against the appropriate blank. Acid and water blanks were included to correct for background absorbance. Two technical replicates were measured for each biological replicate to improve analytical precision. Phytic acid concentration was calculated using a standard curve prepared from PA standards ranging from 0 to 1.0 mg mL^-1^ in 0.65 M HCl. The standard curve showed good linearity, and PA content was expressed on a dry-weight basis (mg g^-1^ DW).

### Statistical analysis and estimation of genetic parameters

Analysis of variance (ANOVA), descriptive statistics, variance components, and broad-sense heritability were estimated for kernel PA content using the R package *variability* (Popat et al., 2020). Single-environment analyses were performed separately for AP2021 (E1) and PUN2022 (E2) using replication and genotype effects. The following linear model was used: Yᵢ_j_ = μ+Rᵢ+G_j_+εᵢ_j_; where Yᵢ_j_ is the observed PA value of the *j*th genotype in the *i*th replication, μ is the overall mean, Rᵢ is the effect of the *i*th replication, G_j_ is the effect of the *j*th genotype, and εᵢ_j_ is the residual error. Genotypic effects were tested against the residual mean square using the F-test: F = MS_G_/MS_E_; where MS_G_ is the genotype mean square and MS_E_ is the residual mean square. Statistical significance was assessed at P < 0.05, P < 0.01, and P < 0.001. Best linear unbiased predictions (BLUPs) were estimated using META-R to obtain adjusted genotypic values for downstream genome-wide association analysis (Alvarado et al., 2020). Across-environment BLUPs were estimated under a mixed-model framework in which environment and replication effects were included as design effects, while genotype and genotype × environment interaction were fitted as random effects. The combined-environment model was expressed as: Yᵢ_j__k_ = μ+Eᵢ+R_k_(ᵢ)+G_j_+GEᵢ_j_+εᵢ_j__k_; where Yᵢ_j__k_ is the observed PA value of the *j*th genotype in the *k*th replication within the *i*th environment, μ is the grand mean, Eᵢ is the effect of the *i*th environment, R_k_(ᵢ) is the effect of the *k*th replication nested within the *i*th environment, G_j_ is the random effect of the *j*th genotype, GEᵢ_j_ is the random genotype × environment interaction effect, and εᵢ_j__k_ is the residual error. Genotypic, genotype × environment interaction, and residual effects were assumed to follow normal distributions with mean zero and variances σ^2^_G_, σ^2^_GE_, and σ^2^_e_, respectively. The resulting BLUPs represented adjusted genotypic performance after accounting for environmental, replication, and genotype × environment interaction effects, following standard mixed-model approaches for plant breeding and multi-environment trials (Piepho et al., 2008). For the combined analysis, broad-sense heritability on an entry-mean basis was calculated as H^2^ = σ^2^_G_/[σ^2^_G_ + (σ^2^_GE_/e) + (σ^2^_e_/re)], where e is the number of environments and r is the number of replications per environment.

Descriptive statistics, including maximum, minimum, grand mean, standard error of mean (SEm), critical difference (CD) at 5% and 1%, variance components, coefficients of variation, broad-sense heritability, and genetic advance, were estimated for kernel PA content in each environment. Residual or environmental variance was estimated as: σ^2^e = MS_E_; Genotypic variance was estimated as: σ^2^_G_ = (MS_G_-MS_E_)/r; Phenotypic variance was estimated as: σ^2^_P_ = σ^2^_G_+σ^2^_e_; where σ^2^_e_ is the residual or environmental variance, σ^2^_G_ is the genotypic variance, σ^2^_P_ is the phenotypic variance, MS_G_ is the genotype mean square, MS_E_ is the residual mean square, and r is the number of replications. Broad-sense heritability on a plot basis was calculated as: H^2^ = σ^2^_G_/σ^2^_P_. The genotypic, phenotypic, and environmental coefficients of variation were calculated as: GCV(%) = (√σ^2^_G_/X̄)×100; PCV(%) = (√σ^2^_P_/X̄)×100; ECV (%) = (√σ^2^_e_/X̄)×100; where X̄ is the grand mean. Genetic advance was estimated as: GA = k × √σ^2^_P_ × H^2^; where k is the selection intensity constant and H^2^ is broad-sense heritability. Phenotypic frequency distributions for AP2021 (E1), PUN2022 (E2), and the pooled dataset (E3) were visualized in R using *rMVP* (v1.4.6) as density-overlaid histograms.

### DNA extraction, sequencing-based genotyping, and SNP filtering

At the V4 stage, young leaf tissues were collected from plants of each genotype and pooled to obtain a representative composite sample for each QPM inbred line. Total genomic DNA was extracted from 249 QPM inbred lines using the cetyltrimethylammonium bromide (CTAB) method (Doyle and Doyle, 1990). The quality and integrity of the extracted DNA were assessed using 0.8% agarose gel electrophoresis, while DNA concentration and purity were determined using a NanoDrop™ spectrophotometer. High-throughput sequencing-based genotyping was performed by NGB Diagnostics Pvt. Ltd., Noida, India. Genomic DNA (500 ng per sample) was used for library preparation using the NEBNext Ultra II DNA Library Preparation Kit for Illumina, with ApeKI used as the restriction enzyme. Following library preparation, indexed libraries were PCR enriched and sequenced on an Illumina NovaSeq 6000 S4 platform using 2 × 151-bp paired-end reads. Sequencing of the 249 QPM inbred lines generated a total of 546.8 million reads, with an average of approximately 2.20 million reads per genotype. Initial demultiplexing of the sequencing pools was performed using CASAVA v1.8.2, after which individual samples were demultiplexed from the pooled data based on barcode sequences using FASTX Toolkit v0.0.13. Read quality was assessed using FastQC v0.11.8, and residual Illumina adapter sequences were rechecked and removed using Trim Galore v0.6.6. Reads were aligned to the *Zea mays* B73 reference genome assembly Zm-B73-REFERENCE-NAM-5.0 (Hufford et al., 2021) using BWA-MEM v0.7.5, and variants were called using GATK v3.8. Variant calling identified 11,525,180 variants. Initial filtering with VCFtools v0.1.17 retained 115,416 chromosomal biallelic SNPs with minor allele frequency (MAF) ≥ 0.05 and genotype calls in at least 10% of samples; no Hardy-Weinberg equilibrium filter was applied. The resulting 115,416-SNP dataset was converted to HapMap format using TASSEL v5.0. For downstream analyses, markers with more than 10% missing data or a heterozygous-call rate greater than 20% were removed. This stricter filtering yielded 26,074 high-quality SNPs for population structure, linkage disequilibrium, and genome-wide association analyses of kernel PA content (Supplementary Table S2).

### Population structure and genetic relatedness analysis

Population structure and genetic relatedness among the inbred lines were assessed from the filtered SNP dataset using Bayesian model-based clustering, principal component analysis (PCA), kinship analysis, and genetic distance-based clustering. For Bayesian clustering, 5,000 pruned SNP markers were analyzed in STRUCTURE v2.3.4 under the admixture model with correlated allele frequencies (Pritchard et al., 2000). Putative genetic clusters (K) were tested from 1 to 10, with three independent runs per K, each comprising 10,000 burn-in iterations followed by 20,000 Markov chain Monte Carlo (MCMC) iterations. The most likely number of subpopulations was determined using the Evanno ΔK method based on the rate of change in the log probability of data across successive K values (Evanno et al., 2005). STRUCTURE output files were parsed in R, and ΔK values, mean LnP(K), cluster composition, and ancestry proportion plots were generated using *tidyverse*, *stringr*, *readr*, *ggplot2*, *RColorBrewer*, and *patchwork* packages. PCA was performed in TASSEL using the filtered genome-wide SNP dataset to summarize major axes of genetic variation among the inbred lines (Bradbury et al., 2007). The first two principal components were visualized in R using *ggplot2*, and the percentage of variance explained by leading components was displayed as a scree plot. Pairwise genetic relatedness was estimated by generating a kinship matrix in TASSEL from genome-wide SNP data, and a genetic distance matrix was generated to assess overall genetic divergence among inbred lines (Bradbury et al., 2007). Kinship and genetic distance matrices were visualized in R as clustered heatmaps using *pheatmap* and *RColorBrewer*. PCA covariates and the kinship matrix were subsequently used, where applicable, to control for population structure and background genetic relatedness in downstream genome-wide association analyses.

### Genome-wide association mapping

Genome-wide association analysis was performed to identify genomic loci associated with kernel PA content in the QPM association panel. Environment-wise and pooled BLUP values for kernel PA content were used as phenotypic inputs for GWAS. To increase the reliability of marker-trait association (MTA) detection and reduce dependence on a single statistical model, GWAS was conducted using multiple complementary approaches implemented in mrMLM v5.0.1, GAPIT v4.1.0, and rMVP v1.4.6. The mrMLM package was used for multi-locus GWAS because multi-locus models can jointly evaluate multiple putative quantitative trait nucleotides (QTNs) and are effective for detecting loci with small to moderate effects in complex quantitative traits (Wang et al., 2016; Zhang et al., 2020). For the mrMLM-family analyses, QTNs with a LOD score ≥3.0 were considered significant. Marker-trait associations detected using mrMLM-based models were further examined using GAPIT and rMVP to assess their support across independent GWAS platforms. In GAPIT, four association models were used: generalized linear model (GLM), mixed linear model (MLM), Fixed and Random Model Circulating Probability Unification (FarmCPU), and Bayesian-information and Linkage-disequilibrium Iteratively Nested Keyway (BLINK). GLM was included as a basic fixed-effect association model, whereas MLM was used to account for both population structure and kinship, thereby reducing spurious associations caused by stratification and relatedness among genotypes (Yu et al., 2006). FarmCPU was included because it uses iterative fixed- and random-effect models to separate marker testing from covariate optimization, improving statistical power while controlling false positives (Liu et al., 2016). BLINK was used because it improves computational efficiency and detection power through Bayesian information criterion-based marker selection and linkage disequilibrium-based filtering of associated markers (Huang et al., 2019). For GAPIT analyses, associations with nominal P < 0.001 were retained as suggestive signals for cross-model comparison. With 26,074 SNPs, the Bonferroni-corrected genome-wide significance threshold at α = 0.05 was P < 1.92 × 10^−6^; none of the GAPIT associations exceeded this threshold. The rMVP package was additionally used with GLM, MLM, and FarmCPU models because it provides a memory-efficient platform for large-scale GWAS and visualization (Yin et al., 2021).

In GAPIT, the first three principal components (PC1-PC3) were included as covariates in the GLM, MLM, FarmCPU, and BLINK models (PCA.total = 3). In rMVP, five PCs were initially calculated (pcs.keep = 5), but only PC1-PC3 were included as covariates in the GLM, MLM, and FarmCPU models; the rMVP MLM additionally incorporated a VanRaden kinship matrix. In the mrMLM-family analyses, no external PCA covariate matrix was supplied. PC1, PC2, and PC3 explained 6.67%, 3.38%, and 3.02% of genotypic variation, respectively. These three PCs were selected as a parsimonious adjustment for the leading axes of population structure based on inspection of the PCA distribution and scree profile; no formal eigenvalue cutoff was applied. Kinship/relatedness was otherwise handled according to the framework of each GWAS model. Manhattan plots were generated to visualize genome-wide MTAs. A representative quantile-quantile (QQ) plot from the GAPIT analyses, including GLM, MLM, FarmCPU, and BLINK, was used to assess potential inflation of association test statistics (Supplementary Figure S3). High-confidence MTAs were defined as QTNs detected by one or more mrMLM-family methods and supported by at least one additional GWAS model implemented in GAPIT and/or rMVP. Thus, the high-confidence designation was based primarily on consistent support across complementary GWAS models and platforms rather than on Bonferroni-corrected genome-wide significance in any individual GWAS model. These high-confidence loci were used for downstream LD-based haplotype analysis, candidate gene mining, functional annotation, variant-effect prediction, and *in silico* expression profiling.

### Linkage disequilibrium, allelic effect, and haplotype effect analyses

Genome-wide linkage disequilibrium (LD) decay was estimated using TASSEL v5.0 by calculating pairwise LD as squared allele-frequency correlations (*r* ^2^) among SNP markers with a sliding window of 1,000 SNPs (Bradbury et al., 2007). The TASSEL-generated LD output was further analyzed in R by fitting the Hill-Weir nonlinear regression model to describe the relationship between LD and physical distance (Hill and Weir, 1988). The physical distance at which LD declined to the predefined threshold of *r* ^2^ = 0.10 was considered the LD decay distance. LD blocks surrounding high-confidence MTAs were examined using Haploview v4.2 (Barrett et al., 2005). For each significant SNP, the surrounding genomic region was inspected for LD block formation. Where flanking SNPs formed an LD-defined block, haplotype structure was visualized in Haploview and retained for downstream haplotype effect analysis.

Allelic effect analysis was performed for high-confidence SNPs to determine whether different SNP genotype classes were associated with variation in kernel PA content. For each SNP, genotypes were grouped according to their observed SNP genotype, and genotypes with missing allele calls at the respective SNP were excluded from the analysis. Differences in PA content among genotype classes were tested using one-way analysis of variance based on the pooled dataset (E3). When significant differences were detected, *post hoc* pairwise comparisons were performed to separate genotype-class means. The results were visualized using boxplots showing the distribution of PA content for each SNP genotype class, the number of genotypes per class, ANOVA P values, and compact letter groupings. Genotype classes associated with lower kernel PA content were considered favorable.

For haplotype effect analysis, genotypes were classified according to nucleotide combinations within LD-defined haplotype blocks, and genotypes with missing or ambiguous allele calls within the haplotype-defining SNPs were excluded. Haplotype-wise differences in kernel PA content were evaluated using the pooled dataset (E3). Summary statistics were calculated for each haplotype class, and assumptions for parametric analysis were assessed using the Shapiro-Wilk test for residual normality and Levene’s test for homogeneity of variance (Shapiro and Wilk, 1965; Levene, 1960). Because the residual normality assumption was not satisfied, the Kruskal-Wallis test was used to assess overall differences among haplotype classes, followed by Dunn’s *post hoc* test with adjusted P values for pairwise comparisons (Kruskal and Wallis, 1952; Dunn, 1964; Piepho, 2004). Haplotype classes associated with comparatively lower kernel PA content were considered favorable.

### Candidate gene identification, functional annotation, and variant-effect prediction

High-confidence MTAs retained from the multi-model GWAS were used for candidate-gene identification. For each significant MTA, all annotated genes located within a predefined 100-kb genomic window surrounding the peak SNP (Lü et al., 2025) were retrieved from Ensembl Plants using the *Zea mays* B73 reference genome assembly Zm-B73-REFERENCE-NAM-5.0 (Yates et al., 2022). These genes were initially considered positional genes associated with the corresponding MTA. Functional annotation was performed using gene descriptions, Gene Ontology (GO) terms, predicted protein domains, InterPro annotations, and available information from Ensembl Plants and MaizeGDB. To determine the potential functional consequences of significant SNPs, variant-effect prediction was performed using the Ensembl Variant Effect Predictor (VEP) against the *Zea mays* B73 NAM v5.0 reference genome (McLaren et al., 2016). SNP effects were classified according to their predicted genomic consequences, including missense, synonymous, intronic, 5′UTR, upstream, downstream, and intergenic variants. Candidate genes were prioritized for biological interpretation based on MTA proximity, predicted SNP consequence, functional annotation, and literature support linking them to seed development, phosphorus metabolism, nutrient signaling, storage-reserve biology, or related regulatory processes.

### *In silico* expression analysis of candidate genes

To examine the biological relevance of candidate genes associated with kernel PA content, *in silico* expression analysis was performed using publicly available maize RNA-seq expression datasets available through the MaizeGDB qTeller platform. Candidate gene IDs identified from the Zm-B73-REFERENCE-NAM-5.0 genome assembly were used as input in qTeller, and expression values were retrieved from the B73v5 [NAM Consortium] dataset (Woodhouse et al., 2022). Expression profiles were examined across maize reproductive, seed-development, vegetative, and phosphate-response tissues, with particular emphasis on embryo, endosperm, whole seed, ear primordium, pericarp/aleurone, germinating kernel, leaf, and root tissues, because PA accumulation is closely associated with seed development and phosphorus metabolism (Raboy, 2003; Shi et al., 2007). Expression values were transformed as log_2_(expression +1) to reduce the influence of highly expressed genes and improve visualization of transcript-abundance differences across tissues and developmental stages. The transformed expression matrix was used to generate a heatmap in R using the *ComplexHeatmap* package (Gu et al., 2016). Tissue-specific expression patterns were used to support biological interpretation of candidate genes potentially involved in kernel development, phosphorus allocation, and PA accumulation.

## Results

### Phenotypic variation and genetic variability for kernel phytic acid content

Kernel PA content showed substantial variation among the 249 QPM inbred lines across environments (Supplementary Table S1). In AP2021 (E1), PA content ranged from 2.16 to 4.30 mg g^-1^ DW, with a mean of 2.99 mg g^-1^ DW, whereas in PUN2022 (E2), it ranged from 1.98 to 4.14 mg g^-1^ DW, with a mean of 2.80 mg g^-1^ DW (Supplementary Table S3). The mean ± SD values were 2.99 ± 0.26, 2.80 ± 0.27, and 2.90 ± 0.27 mg g^-1^ DW in E1, E2, and the pooled dataset, respectively. Distribution plots showed continuous variation across E1, E2, and the pooled dataset, with most genotypes clustered around the population mean and a few showing relatively low or high PA values (Figures 1A–C). Analysis of variance revealed highly significant genotypic effects for kernel PA content in both environments (P < 2 × 10^−16^), whereas replication effects were not significant, indicating that the observed variation was primarily attributable to genotypic differences among QPM inbred lines (Supplementary Table S4). Genetic parameter estimates further supported strong genetic control of the trait. Genotypic variance exceeded residual or environmental variance in both E1 and E2, and genotypic and phenotypic coefficients of variation were comparable. Broad-sense heritability on a plot basis was high in both environments, with estimates of 0.92 in E1 and 0.99 in E2, while genetic advance was 0.53 and 0.55, respectively (Supplementary Table S3). In the combined analysis, σ^2^G and σ^2^e were 0.07295 and 0.00262, respectively, whereas σ^2^GE was estimated at zero. Broad-sense heritability on an entry-mean basis was 0.991. The zero σ^2^GE estimate indicates little detectable G × E in this dataset; however, evaluation across additional environments is needed for a more robust estimate of G × E variance. Overall, the continuous phenotypic variation, highly significant genotypic effects, and high heritability indicate that kernel PA content is suitable for genome-wide association analysis in the QPM panel.

**FIGURE 1:**
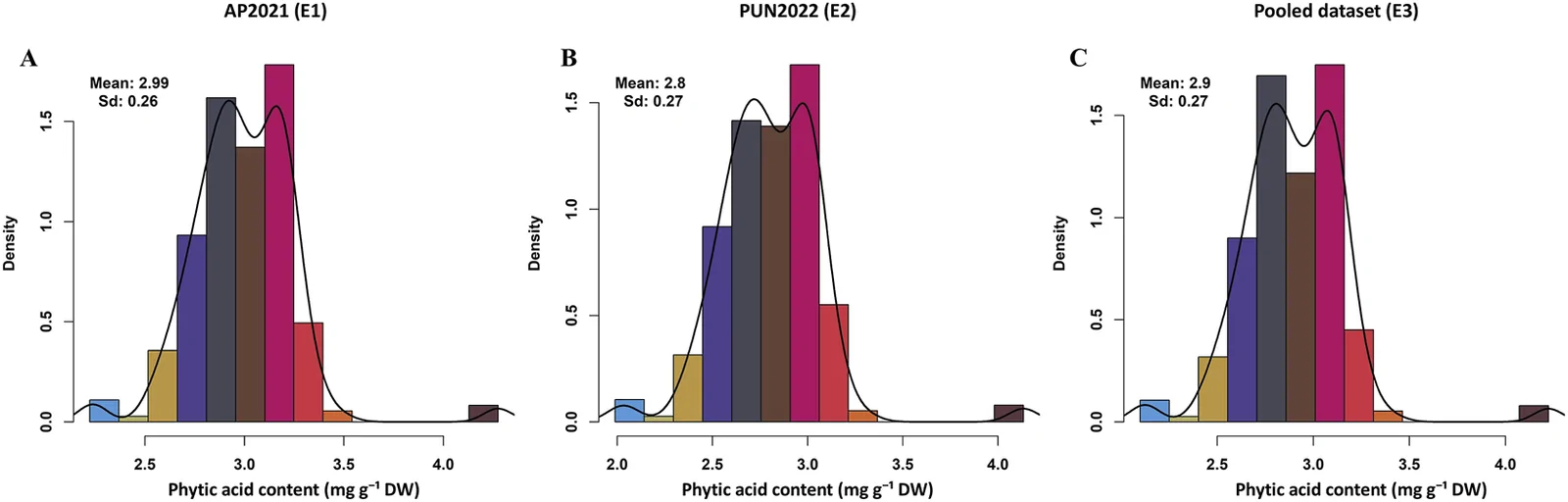
Phenotypic distribution of kernel phytic acid content in the QPM association panel. Distribution of kernel phytic acid content among 249 QPM inbred lines in **(A)** AP2021 (E1), **(B)** PUN2022 (E2), and **(C)** pooled dataset (E3). Mean and standard deviation values are shown within each panel.

### SNP marker dataset, population structure, genetic relatedness, and linkage disequilibrium

Sequencing initially generated 115,416 genome-wide SNP markers across the 249 QPM inbred lines. Subsequent filtering for missing data and heterozygous-call rate reduced the dataset to 26,074 high-quality SNPs across all 10 chromosomes, with 1,791–4,224 SNPs per chromosome and a mean adjacent-marker spacing of approximately 81.1 kb (Supplementary Figure S1). STRUCTURE analysis supported K = 2 as the most likely number of genetic clusters, indicating two inferred genetic groups with a substantial proportion of admixed lines (Figure 2A; Supplementary Figures S4A–C). At K = 2, 53 lines were assigned to Cluster_1, 49 lines to Cluster_2, and 147 lines were classified as admixed, representing 21.29%, 19.68%, and 59.04% of the panel, respectively (Supplementary Figure S4D). Principal component analysis also indicated weak-to-moderate population structure. The first four principal components explained 6.67%, 3.38%, 3.02%, and 2.72% of the total genotypic variation, respectively. The PCA scatter plot showed a largely continuous distribution of genotypes, while the scree plot showed that no single PC explained a large proportion of the variation (Figures 2B,C). The kinship heatmap revealed generally low-to-moderate relatedness among most lines, with small clusters of closely related genotypes (Figure 2D). Together, these results supported model-specific adjustment for population structure and relatedness in the GWAS analyses. Genome-wide LD decayed to *r* ^2^ = 0.10 at 3.7 kb, ranging from 1.8 kb on chromosome six–8.6 kb on chromosome 4 (Supplementary Table S5). Because mean marker spacing exceeded the genome-wide LD decay distance, the marker set provided broad but nonuniform genome coverage; associations in sparsely covered regions may therefore have been missed, and significant loci require further fine mapping.

**FIGURE 2:**
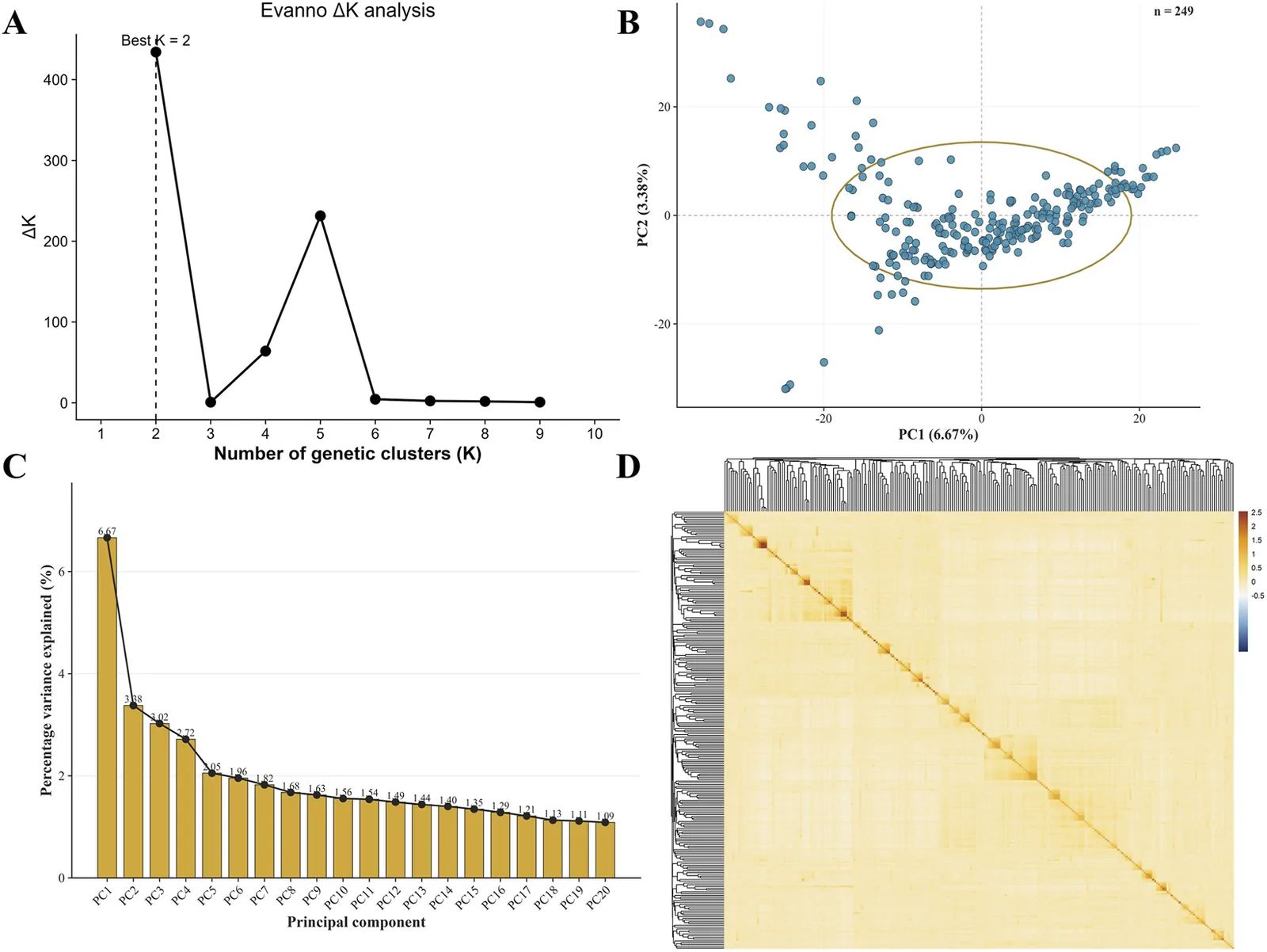
Population structure and genetic relatedness of the 249 QPM inbred lines. **(A)** Delta K statistics showing the most likely number of genetic subpopulations in the association panel. **(B)** Principal component analysis scatter plot showing the distribution of 249 QPM inbred lines based on genome-wide SNP markers. **(C)** Scree plot showing the percentage of genetic variation explained by the first 20 principal components. **(D)** Kinship heatmap showing pairwise genetic relatedness among the inbred lines.

### Genome-wide association mapping for kernel phytic acid content

Genome-wide association mapping identified multiple genomic regions associated with kernel PA content across E1, E2, and the pooled dataset (E3). The mrMLM-based analysis detected 12 significant QTNs distributed across chromosomes 1, 2, 3, 4, 6, and 7, with QTN effects ranging from −0.24 to 0.11 and phenotypic variation explained ranging from 0.1% to 17.7% (Supplementary Figure S2; Supplementary Table S6). These results indicate that kernel PA content is controlled by multiple loci with variable effect sizes.

To improve confidence in marker detection, mrMLM-detected QTNs were compared with associations identified using GAPIT and rMVP models (Supplementary Tables S7, S8). Five QTNs were supported by at least one additional GWAS platform and were retained as high-confidence MTAs: chr1:254454440, chr1:288704637, chr3:236714544, chr4:176947058, and chr7:181099438 (Table 1). These high-confidence MTAs were distributed across chromosomes 1, 3, 4, and 7, with minor allele frequencies ranging from 0.12 to 0.22. Among these loci, chr3:236714544 was the strongest and most consistent association. It was detected in both E1 and E2 and was also retained in the pooled E3 analysis by multiple mrMLM-family methods, including ISIS EM-BLASSO, mrMLM, FASTmrMLM, pKWmEB, FASTmrEMMA, and pLARmEB, with additional support from GAPIT and rMVP. This marker showed the largest effect, with QTN effects ranging from −0.24 to −0.12, LOD scores of 3.2–9.0, -log10(P) values of 3.9–10.0, and phenotypic variation explained ranging from 5.7% to 17.7%. The two chromosome 1 loci, chr1:254454440 and chr1:288704637, were detected by mrMLM in E2 and E3 and were also supported by GAPIT, with chr1:254454440 additionally supported by rMVP. The chromosome four locus, chr4:176947058, was detected by pLARmEB and pKWmEB in E1 and was further supported by both GAPIT and rMVP across datasets. Although this locus showed a smaller mrMLM-derived effect and explained a lower proportion of phenotypic variation, its repeated detection across independent GWAS platforms supported its inclusion as a high-confidence MTA. The chromosome 7 locus, chr7:181099438, was detected by mrMLM in E2 and E3 and was further supported by rMVP across FarmCPU, GLM, and MLM models. Overall, multi-model GWAS identified five high-confidence MTAs for kernel PA content in the QPM panel, with chr3:236714544 representing the strongest candidate locus. These loci were retained for downstream allelic effect analysis, LD-based haplotype analysis, candidate-gene identification, variant-effect prediction, and expression profiling.

**TABLE 1:** High-confidence marker–trait associations for kernel phytic acid content in Quality Protein Maize identified by multi-model GWAS across all three datasets.

QTN	Marker position (bp)	mrMLM-family method(s)	GAPIT model(s)	rMVP model(s)	mrMLM QTN effect	mrMLM LOD score	mrMLM -log10(P)	mrMLM *R* ^2^ (%)	MAF
chr1:254454440	254454440	mrMLM	BLINK, FarmCPU, GLM, MLM	FarmCPU, GLM, MLM	−0.12	4.2–5.7	4.9–6.5	7.9–8.3	0.13
chr1:288704637	288704637	mrMLM	BLINK, FarmCPU, GLM, MLM	-	−0.12 to −0.11	3.5–3.6	4.2–4.3	6.8–7.4	0.12
chr3:236714544	236714544	ISIS EM-BLASSO, mrMLM, pKWmEB, FASTmrMLM, FASTmrEMMA, pLARmEB	BLINK, FarmCPU, GLM, MLM	FarmCPU, GLM, MLM	−0.24 to −0.12	3.2–9	3.9–10	5.7–17.7	0.14
chr4:176947058	176947058	pLARmEB, pKWmEB	BLINK, FarmCPU, GLM, MLM	FarmCPU, GLM, MLM	−0.04 to −0.01	3.4–5.6	4.1–6.5	0.1–3.3	0.22
chr7:181099438	181099438	mrMLM	-	FarmCPU, GLM, MLM	−0.12	4.1–4.5	4.8–5.3	7.4–7.8	0.12

QTN, quantitative trait nucleotide; LOD, logarithm of odds; *R* ^2^ = phenotypic variance explained; MAF, minor allele frequency. High-confidence MTAs were defined as mrMLM-family QTNs supported by at least one additional GWAS platform (GAPIT and/or rMVP). A dash (−) indicates that the corresponding locus was not detected by that platform. Ranges indicate values observed across datasets and/or mrMLM-family methods.

### Allelic and haplotype effects of significant marker-trait associations

Allelic effect analysis of the five high-confidence MTAs revealed significant differences in kernel PA content among SNP genotype classes in the pooled E3 dataset (Figures 3A–E). All five SNPs showed significant allelic effects, with ANOVA P values ranging from 7.31 × 10^−6^ to 0.0177 (Figures 3A–E). The strongest allelic differentiation was observed for chr3:236714544 (P = 7.31 × 10^−6^), where the AA genotype showed the highest PA content, the GA genotype showed an intermediate value, and the GG genotype showed the lowest value. Significant allelic effects were also observed for chr1:254454440 (P = 0.00101), chr1:288704637 (P = 0.0177), chr4:176947058 (P = 0.00775), and chr7:181099438 (P = 0.000642). Across these loci, the lower-phytic-acid genotype classes were GG at chr1:254454440, GG at chr1:288704637, GG at chr3:236714544, AA at chr4:176947058, and TT/TC at chr7:181099438 (Figure 3F).

**FIGURE 3:**
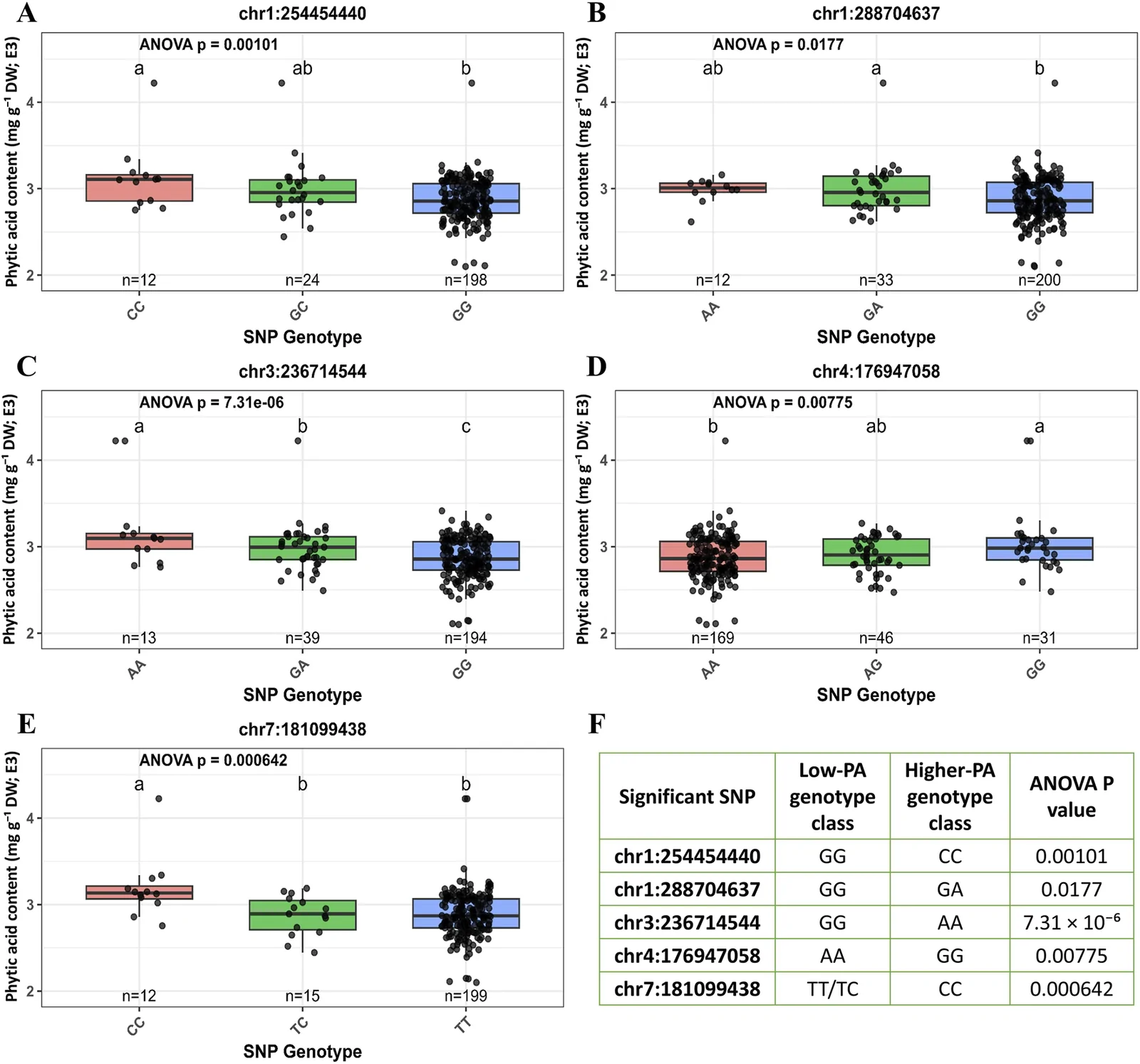
Allelic effects of high-confidence MTAs associated with kernel phytic acid content. Boxplots showing differences in kernel phytic acid content among SNP genotype classes for **(A)** chr1:254454440, **(B)** chr1:288704637, **(C)** chr3:236714544, **(D)** chr4:176947058, and **(E)** chr7:181099438 in the pooled dataset (E3). Different letters indicate significant differences among genotype classes based on *post hoc* comparison. Panel **(F)** summarizes low- and higher-phytic-acid genotype classes for each significant SNP.

LD-based haplotype analysis was performed for genomic regions surrounding the high-confidence MTAs. Among the examined regions, only the chromosome four locus surrounding chr4:176947058 formed a distinct three-SNP LD block, comprising SNPs at 176946831, 176947058, and 176947073 bp, with strong pairwise LD values ranging from 0.93 to 0.98 (Figure 4A). Three haplotypes were identified: Hap1/AAC, Hap2/GAC, and Hap3/GGT. After excluding genotypes with missing or ambiguous allele calls, 157 genotypes were retained for haplotype analysis (Figure 4B). Hap2/GAC was the most frequent haplotype (n = 97), followed by Hap1/AAC (n = 33) and Hap3/GGT (n = 27). Haplotype-wise comparison showed significant differences in kernel PA content among the three haplotypes in the pooled E3 dataset. Hap2/GAC showed the lowest mean PA content, Hap3/GGT showed the highest mean, and Hap1/AAC showed an intermediate value (Figure 4C). The Kruskal-Wallis test detected a significant overall haplotype effect (P = 0.0381), and Dunn’s *post hoc* test indicated a significant difference between Hap2/GAC and Hap3/GGT (Figure 4B).

**FIGURE 4:**
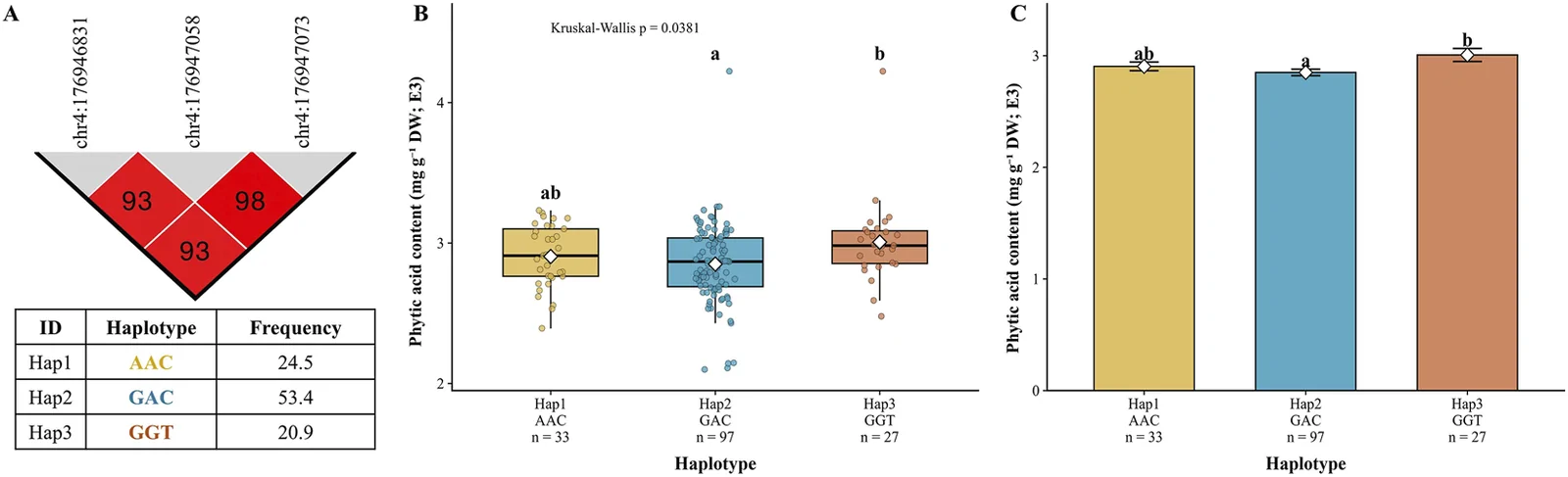
LD-based haplotype analysis of the chromosome four locus associated with kernel phytic acid content. **(A)** Linkage disequilibrium block surrounding chr4:176947058 and haplotypes defined by three linked SNPs. **(B)** Haplotype-wise distribution of kernel phytic acid content in the pooled dataset (E3). **(C)** Mean kernel phytic acid content ±SE for each haplotype class. Different letters indicate significant differences among haplotypes based on *post hoc* comparison.

### Functional annotation, variant-effect prediction, and expression profiling of candidate genes

Candidate-gene mining within 100-kb windows surrounding the five high-confidence MTAs identified 30 genes, of which seven were prioritized as putative candidates based on functional annotation and literature-supported biological relevance (Supplementary Table S9). Two peak MTAs were located within gene bodies: chr3:236714544 within *Zm00001eb163880* and chr4:176947058 within *Zm00001eb191620*. The other prioritized candidates were *Zm00001eb049830* near chr1:254454440, *Zm00001eb058940* near chr1:288704637, *Zm00001eb163850* and *Zm00001eb163870* within the chr3 interval, and *Zm00001eb330080* near chr7:181099438 (Table 2). Functional annotation indicated that these genes represented diverse biological categories, including transcriptional regulation, phosphorylation-dependent signaling, phosphate transport, seed development, and lipid/protein storage-related processes. Specifically, *Zm00001eb049830* encoded a zinc finger/RING-type domain-containing protein, whereas *Zm00001eb058940* and *Zm00001eb330080* encoded protein kinase domain-containing proteins with ATP-binding functions. At the chr3:236714544 locus, *Zm00001eb163850* encoded a WRKY domain-containing transcriptional regulator, *Zm00001eb163870* encoded a triose phosphate/phosphate translocator domain-containing protein, and *Zm00001eb163880* encoded a plant non-specific lipid-transfer protein/seed-storage helical domain-containing protein. The chromosome four gene-body candidate *Zm00001eb191620* encoded a PB1 domain-containing protein (Table 2).

**TABLE 2:** Functional annotation of candidate genes associated with kernel phytic acid content in Quality Protein Maize.

QTN name	Gene stable ID	Gene start (bp)	Gene end (bp)	GO term name	GO term definition	Interpro description
chr1254454440	*Zm00001eb049830*	254500702	254507995	Histone H3K9 demethylase activity	Catalysis of the removal of a methyl group from a modified lysine residue at position 9 of the histone H3 protein	Zinc finger, RING-type
chr1288704637	*Zm00001eb058940*	288661070	288668582	ATP binding	Binding to ATP, adenosine 5′-triphosphate, a universally important coenzyme and enzyme regulator	Protein kinase domain
chr3236714544	*Zm00001eb163850*	236635882	236639269	Regulation of DNA-templated transcription	Any process that modulates the frequency, rate or extent of cellular DNA-templated transcription	WRKY domain
chr3236714544	*Zm00001eb163870*	236706749	236710244	Membrane	A lipid bilayer along with all the proteins and protein complexes embedded in it and attached to it	Triose phosphate/Phosphate translocator
chr3236714544	*Zm00001eb163880*	236713751	236714677	Lipid binding/transport	Binding to a lipid./The directed movement of lipids into, out of or within a cell, or between cells, by means of some agent such as a transporter or pore. Lipids are compounds soluble in an organic solvent but not, or sparingly, in an aqueous solvent	“Plant non-specific lipid-transfer protein” and “par allergen and bifunctional inhibitor/plant lipid transfer protein/seed storage helical domain”
chr4176947058	*Zm00001eb191620*	176945621	176948051	Protein binding	Binding to a protein	PB1 domain
chr7181099438	*Zm00001eb330080*	181087293	181091951	ATP binding	Binding to ATP, adenosine 5′-triphosphate, a universally important coenzyme and enzyme regulator	Protein kinase domain

*In silico* expression profiling revealed distinct tissue- and developmental-stage-specific expression patterns among the candidate genes (Supplementary Table S10; Figure 5). *Zm00001eb163880* showed the strongest expression among the candidates, with high transcript abundance in whole seed tissues, particularly during early seed developmental stages, as well as in germinating kernels, pericarp/aleurone, and phosphate-treated root tissues. *Zm00001eb191620* showed elevated expression in embryo tissues, particularly at 16 and 38 days after pollination, and was also expressed in whole seed, germinating kernel, and pericarp/aleurone tissues. *Zm00001eb330080* showed moderate expression across several seed-related tissues, including endosperm, whole seed, and germinating kernels, and was also expressed in phosphate-treated root tissues. Among the chromosome 1 candidates, *Zm00001eb049830* showed prominent expression in developing embryo and ear primordium tissues, whereas *Zm00001eb058940* showed lower but detectable expression in embryo, whole seed, ear primordium, and phosphate-treated root tissues. At the chr3 locus, *Zm00001eb163870* showed moderate expression in whole seed, germinating kernel, ear primordium, and phosphate-response tissues (Figure 5). *Zm00001eb163850* showed no detectable expression across the selected qTeller tissues and was therefore not visualized in the heatmap (Supplementary Table S10).

**FIGURE 5:**
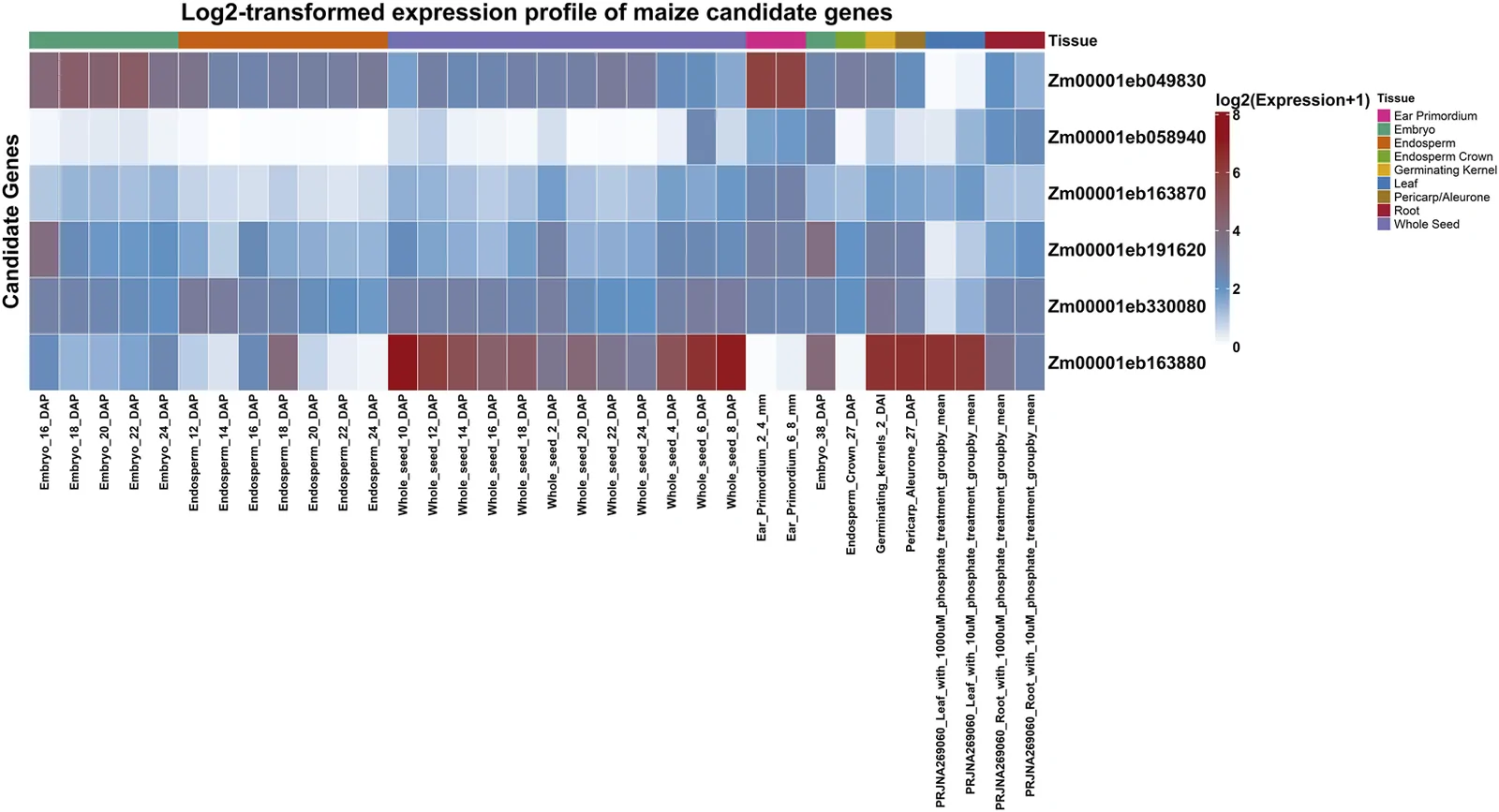
Expression profiles of candidate genes associated with kernel phytic acid content. Heatmap showing log_2_(expression +1)-transformed expression profiles of candidate genes across selected maize seed-development, reproductive, and phosphate-response tissues. Expression values were retrieved from the MaizeGDB qTeller B73v5 dataset, and tissue classes are indicated by the color annotation bar.

Variant-effect prediction clarified the direct genomic consequences of the five high-confidence MTAs. The strongest and most consistent association, chr3:236714544, was located within *Zm00001eb163880* and was predicted as a missense variant, making *Zm00001eb163880* the leading candidate gene. The chr4:176947058 marker was located within *Zm00001eb191620* but was predicted as a synonymous variant; therefore, this locus was interpreted as a genic haplotype-associated signal rather than direct evidence for a protein-altering causal variant. VEP annotated chr1:254454440 as a 5′ UTR variant of *Zm00001eb049810*, chr7:181099438 as an intronic variant within *Zm00001eb330100*, and chr1:288704637 as intergenic. Notably, the VEP-hit genes *Zm00001eb049810* and *Zm00001eb330100* differed from the nearby positional candidates *Zm00001eb049830* and *Zm00001eb330080*, respectively. The positional candidates were prioritized based on MTA proximity, functional annotation, and expression support, whereas the VEP-hit genes lacked detectable expression in the tissues examined. Integrating positional, functional, variant-effect, and expression evidence identified *Zm00001eb163880* as the strongest candidate at chr3:236714544, supported by a predicted missense variant and strong seed-related expression. *Zm00001eb191620* and *Zm00001eb330080* were also prioritized as plausible candidates based on their genomic positions, functional annotations, and expression in seed- or phosphate-responsive tissues. These genes represent priority targets for functional validation of kernel PA regulation in maize.

## Discussion

### Natural variation for kernel phytic acid in QPM and its breeding relevance

Reducing kernel PA remains an important objective for improving the nutritional value of maize grain because phytate is the major phosphorus-storage compound in seeds but strongly reduces the bioavailability of Fe, Zn, Ca, Mg, and other mineral cations in human and monogastric diets (Raboy, 2003; Bohn et al., 2008). This issue is particularly relevant in QPM, where the nutritional limitation of poor protein quality has already been addressed through the *opaque2* allele and endosperm modifier genes that restore kernel hardness while enhancing lysine and tryptophan content (Mertz et al., 1964; Vasal, 2000; Prasanna et al., 2001; Paul et al., 2026). However, improved protein quality alone does not remove the anti-nutritional burden imposed by phytate. Thus, combining QPM with reduced kernel PA represents a logical next step toward maize biofortification aimed at improving both protein quality and mineral bioavailability.

Classical low-phytic-acid (*lpa*) mutants in maize, including *lpa1-1* and *lpa2-1*, demonstrated that seed phytate can be genetically reduced, often with a corresponding increase in inorganic phosphorus (Raboy et al., 2000; Raboy, 2003). However, their direct use in breeding has often been constrained by pleiotropic effects on seed viability, germination, seedling vigor, kernel development, and agronomic performance (Raboy et al., 2000; Shi et al., 2007; Cominelli et al., 2020). These trade-offs are biologically understandable because phytate is not merely an anti-nutritional compound; it is also a major seed phosphorus and mineral reserve required during germination and early seedling establishment (Carelli et al., 2025). In this context, naturally occurring allelic variation within QPM germplasm is especially valuable because moderate-effect alleles may allow incremental PA reduction without severely disrupting essential seed phosphorus-storage functions.

Physical comparison with the classical *lpa* loci further distinguishes the natural associations detected here from known severe low-phytate mutations. In the B73 NAM v5 reference assembly, *lpa1/ZmMRP4* (*Zm00001eb003490*), which encodes an MRP/ABCC transporter, is located at chr1:9,282,200–9,289,007, whereas *lpa2/ZmITPK1* (also reported as *ZmIpk*; *Zm00001eb024800*) is located at chr1:103,263,448–103,264,980; these genes are the established causal loci underlying *lpa1* and *lpa2*, respectively (Shi et al., 2003; Shi et al., 2007). The two chromosome 1 MTAs identified here, chr1:254454440 and chr1:288704637, are therefore well separated from these classical loci, occurring approximately 245.17 and 279.42 Mb from *lpa1* and 151.19 and 185.44 Mb from *lpa2*, respectively. The remaining three MTAs occur on chromosomes 3, 4, and 7. In addition, none of the five associations is located near the chromosome 10 *ZmIPK1* locus (*Zm00001eb432760*; ∼148.89 Mb), which encodes an inositol-pentakisphosphate 2-kinase involved in the terminal phosphorylation of InsP_5_ to InsP_6_; maize contains more than one *IPK1* paralog (Sun et al., 2007). Collectively, these positional comparisons show that the loci identified here are distinct from the classical *lpa1* and *lpa2* genes as well as the chromosome 10 *ZmIPK1* locus considered here, supporting the view that they represent additional natural variation contributing to kernel PA accumulation.

The continuous distribution of kernel PA content observed among the 249 QPM inbred lines supports the quantitative nature of this trait. The absence of clear phenotypic classes indicates that PA accumulation in this panel is unlikely to be governed by a single major segregating mutation; rather, it reflects the cumulative action of multiple loci with small to moderate effects. The high broad-sense heritability estimates in both environments further indicate that genetic variance contributed strongly to the observed phenotypic variation, making selection feasible. These findings are consistent with previous maize studies showing that grain PA concentration is quantitatively controlled and influenced by both genotype and environment (Huang, 2013; Pramitha et al., 2020). Comparable polygenic architectures have also been reported for grain PA in wheat, mungbean, and sorghum (Wen et al., 2022; Sinha et al., 2023; Shaibu et al., 2025). The broad phenotypic range in the present QPM panel is therefore important from a breeding perspective, as it demonstrates that useful variation for PA reduction already exists within a nutritionally improved maize background and can be exploited without first introducing severe *lpa* mutant alleles.

### Multi-model GWAS identifies robust loci for kernel phytic acid content

The reliability of association mapping depends on adequately controlling population structure and relatedness, especially in breeding-derived diversity panels (Saini et al., 2025). In the present QPM panel, STRUCTURE supported two genetic clusters, but the high admixture, continuous PCA distribution, genetic distance clustering, and generally low-to-moderate kinship indicated weak-to-moderate population differentiation. Therefore, PC1-PC3 were included as covariates in the GAPIT and rMVP models, whereas the mrMLM-family analyses were conducted without external PC covariates. This model-specific adjustment was used to reduce spurious associations due to stratification and relatedness. A major strength of the present study is the use of complementary GWAS approaches rather than reliance on a single model. GLM provides a baseline association scan but can be vulnerable to false positives when structure is not sufficiently corrected. MLM improves control of relatedness, whereas FarmCPU and BLINK increase detection power by iteratively separating marker testing from covariate optimization and reducing confounding among linked markers (Yu et al., 2006; Liu et al., 2016; Huang et al., 2019). Multi-locus mrMLM approaches are particularly useful for quantitative traits because they can model multiple putative quantitative trait nucleotides simultaneously and improve the detection of loci with moderate effects (Wang et al., 2016; Zhang et al., 2020; Gudi et al., 2023).

Retaining MTAs supported across complementary GWAS packages and models provided stronger evidence than relying on any single-model result alone. This cross-model support is particularly important for nutritional traits such as PA, where effect sizes may be moderate and biological interpretation depends on distinguishing robust loci from model-specific signals. The combined use of mrMLM, GAPIT, and rMVP, together with GLM, MLM, FarmCPU, BLINK, and multi-locus approaches, increased confidence in the retained MTAs while reducing dependence on a single statistical framework. The filtered marker set, LD decay estimates, and correction for population structure and kinship further provided an appropriate basis for candidate-gene interpretation and haplotype analysis. Among the five high-confidence MTAs, chr3:236714544 emerged as the strongest association for kernel PA content. Its detection in both E1 and E2 and retention in the pooled E3 analysis, together with support from multiple GWAS approaches, strong allelic differentiation, and the highest phenotypic variation explained, prioritized the chromosome 3 region around 236.7 Mb as a major candidate interval in this QPM panel. Although GWAS resolution can be influenced by local LD, marker density, allele frequency, and population history (Xiao et al., 2017; Saini et al., 2022), these findings provide strong evidence for this region as a key locus associated with kernel PA variation. Further validation will help refine the associated interval and support its future use in breeding.

Comparison with the earlier maize QTL study of Huang (2013) suggests substantial positional novelty of the loci identified here. Huang reported PA-associated regions on chromosomes 1, 2, 4, 6, and 7, including a chromosome four bin 4.01 QTL detected under both low- and high-N conditions. Because these QTL were defined using legacy SSR markers and genetic bins, comparison with NAM v5 positions is necessarily approximate. The present chromosome 1 MTAs at 254.45 and 288.70 Mb are ∼41.25 and 75.50 Mb from the *bnlg1556*-associated NAM v5 anchor near 213.20 Mb, while no chromosome 3 PA QTL was reported by Huang, supporting the novelty of the major chr3:236714544 locus. On chromosome 7, chr7:181099438 is ∼15.62 Mb from the *umc1710*-associated anchor near 165.47 Mb, although correspondence with the historical QTL interval cannot be resolved precisely. Given the QPM background of the panel, we also considered possible linkage with *opaque2* (*Zm00001eb301570*); however, its location at ∼10.95 Mb is ∼170 Mb from chr7:181099438, making local linkage unlikely (Hufford et al., 2021; Chen et al., 2023). The chromosome four association at 176.95 Mb also appears distinct from the previously reported bin 4.01 QTL. Overall, the present GWAS extends the previously reported maize PA QTL landscape, although differences in genetic background, marker density, and mapping resolution should be considered when comparing studies.

### Candidate genes and haplotypes suggest broader regulation of seed phosphorus allocation

The candidate-gene evidence at the chromosome 3 locus provides a strong biological basis for prioritization. *Zm00001eb163880* is the leading candidate because chr3:236714544 lies within its gene body and is predicted by VEP as a missense variant. This distinction is important because a missense variant may alter amino-acid properties or protein function but does not necessarily truncate the encoded protein. *Zm00001eb163880* encodes a plant non-specific lipid-transfer protein/seed-storage helical domain-containing protein and shows strong expression in seed-related tissues, including whole seed, pericarp/aleurone, germinating kernels, and phosphate-treated root tissues. Although non-specific lipid-transfer proteins are not canonical enzymes of the inositol phosphate pathway, they are frequently associated with seed tissues, lipid movement, membrane processes, and storage-related functions (Kader, 1996; Boutrot et al., 2008; Wang et al., 2015). These functions could plausibly influence the cellular environment in which phytate is deposited, particularly because phytate accumulates in specialized seed tissues and storage compartments. Thus, the combined evidence from GWAS consistency, allelic effect, predicted missense consequence, and seed-related expression makes *Zm00001eb163880* the most compelling candidate gene in this study.

Two additional genes at the chromosome 3 interval provide useful biological context. *Zm00001eb163870* encodes a triose phosphate/phosphate translocator domain-containing protein, which is relevant because plastidial phosphate translocators coordinate carbon and phosphate exchange between plastids and the cytosol and may influence phosphate partitioning during seed filling (Flügge et al., 2011). Such transport processes could affect the availability and allocation of phosphorus and carbon skeletons during kernel development, thereby indirectly influencing final PA accumulation (Wang et al., 2014). However, because the peak chr3 SNP is not supported as a functional coding variant within this gene, *Zm00001eb163870* may be considered a secondary candidate. *Zm00001eb163850* encodes a WRKY-domain transcription regulator, a class of proteins widely involved in transcriptional regulation, stress responses, development, and nutrient signaling (Rushton et al., 2010; Wang et al., 2014). Its lack of detectable expression in the selected seed and phosphate-response tissues, however, makes it a lower-priority candidate for kernel PA variation in this study. Therefore, while all three genes inform the chromosome 3 interval, *Zm00001eb163880* remains the strongest candidate based on the combined positional, functional, variant-effect, and expression evidence.

The chromosome four locus containing chr4:176947058 illustrates the value of haplotype analysis for interpreting moderate-effect GWAS signals. Although this locus was weaker than the major chromosome 3 association, chr4:176947058 was located within *Zm00001eb191620* and formed part of a strong three-SNP LD block. This block defined haplotypes with contrasting PA levels, with Hap2/GAC associated with lower PA content and Hap3/GGT with higher PA content, indicating that the local genic haplotype background captured useful variation beyond the single peak SNP. Such moderate-effect haplotypes may be valuable for allele pyramiding, enabling gradual PA reduction while minimizing seed-performance penalties often associated with extreme low-phytate mutations. *Zm00001eb191620* encodes a PB1 domain-containing protein and showed elevated expression in developing embryo tissues, particularly at 16 and 38 days after pollination. PB1 domains mediate protein-protein interactions in regulatory networks, including auxin-response factor modules involved in development and tissue differentiation (Guilfoyle and Hagen, 2007; Korasick et al., 2014; Wang et al., 2014). Because phytate deposition in maize is closely associated with embryo and aleurone tissues (Carelli et al., 2025), an embryo-expressed PB1 domain-containing gene within this haplotype block is a biologically plausible candidate for influencing PA accumulation indirectly through seed-development or nutrient-partitioning processes. The broader involvement of PB1/ARF-related regulatory modules in plant development and nutrient-response pathways further supports the relevance of this locus (Guilfoyle, 2015). However, chr4:176947058 was predicted as a synonymous variant within *Zm00001eb191620*; therefore, this association should be interpreted as a genic haplotype-supported signal rather than direct evidence for a protein-altering causal variant. Independent validation will be required before this haplotype can be deployed in breeding.

The remaining loci on chromosomes 1 and 7 represent plausible secondary candidate regions associated with regulatory and signaling processes. The prioritized chromosome 1 candidate, *Zm00001eb049830*, encodes a zinc finger/RING-type domain-containing protein, a class of proteins involved in transcriptional regulation, plant development, and ubiquitin-mediated protein turnover (Peng et al., 2007; Huang et al., 2022; Ye and Tang, 2025). The additional candidates *Zm00001eb058940* on chromosome 1 and *Zm00001eb330080* on chromosome 7 encode kinase-domain proteins, suggesting potential roles in phosphorylation-dependent signaling. Although none of these genes can currently be assigned a direct function in PA biosynthesis, protein kinases broadly regulate nutrient signaling, development, and metabolism (Hrabak et al., 2003), while inositol phosphate metabolism is closely linked to phosphate homeostasis and phytate accumulation (Kuo et al., 2014). These loci may therefore influence kernel PA indirectly through regulatory networks governing phosphorus signaling, seed development, or nutrient transport and storage. Comparable PA association studies in other crops have also identified diverse regulatory, transport, and signaling candidates. In wheat, Wen et al. (2022) identified six PA associations and highlighted candidates including ABC-2 transporter and glycosyltransferase genes; four favorable alleles collectively reduced mean grain PA from 1.21% to 1.13%. In mungbean, five PA-associated SNPs explained 15.4%–18.7% of phenotypic variance, with candidate genes encoding a serine/threonine protein phosphatase and a phosphatidylinositol kinase (Sinha et al., 2023). Similarly, Shaibu et al. (2025) identified four PA-associated SNPs in sorghum and candidate genes including one encoding a receptor kinase. Although these candidates do not imply direct orthology with the maize genes identified here, their diverse transport, signaling, and regulatory functions broadly parallel the candidate-gene patterns observed in the present study.

A broader interpretation from this study is that the retained loci did not primarily point to canonical PA biosynthetic genes, but instead highlighted candidates related to seed development, phosphate allocation, carbon-phosphate exchange, protein kinase signaling, transcriptional regulation, and storage-reserve biology. This agrees with the known biology of phytate accumulation: PA is synthesized during seed development and stored mainly in embryo- and aleurone-associated tissues as mineral phytate salts, where it serves as a phosphorus and mineral reserve for germination (Raboy, 2003; Shi et al., 2007). Severe disruption of core PA transport or biosynthesis can strongly reduce phytate but may also compromise seed performance, as observed in several *lpa* mutants (Raboy et al., 2000; Cominelli et al., 2020; Colombo et al., 2020). By contrast, the loci identified here may represent natural regulatory or partitioning variants that fine-tune final PA content without abolishing essential phosphorus-storage functions. This model is particularly attractive for QPM improvement, where nutritional enhancement must be balanced with kernel hardness, seed vigor, and agronomic performance.

### Implications for QPM biofortification and future validation

From a breeding standpoint, the favorable genotype classes and haplotypes identified in this study provide promising starting points for genomics-assisted reduction of PA in QPM. The low-PA genotype class at chr3:236714544 and the Hap2/GAC haplotype on chromosome four could be converted into breeder-friendly markers after validation. Because each locus appears to have a moderate rather than extreme effect, pyramiding favorable alleles across loci may be more practical than introgressing severe *lpa* mutations. Such a strategy could reduce anti-nutritional burden while preserving the seed phosphorus reserves needed for germination and early growth (Colombo et al., 2020). Marker-assisted selection could be useful for major and validated loci such as chr3:236714544, whereas genomic selection may be appropriate for capturing the broader polygenic component of PA variation across the QPM breeding pool.

However, marker deployment should proceed only after independent validation in diverse QPM and non-QPM backgrounds. It will also be essential to evaluate whether reduced PA is accompanied by desirable changes in inorganic phosphorus, total phosphorus, Fe and Zn concentration, mineral bioavailability, seed germination, seedling vigor, kernel hardness, endosperm modification, grain yield, and overall agronomic performance. Without these complementary measurements, PA reduction alone cannot be assumed to translate into improved nutritional value or field suitability (Gupta et al., 2015). This is particularly important in QPM, where maintenance of hard, vitreous endosperm and acceptable agronomic performance is essential for adoption (Maqbool et al., 2021). Future breeding strategies should therefore treat low PA as one component of a broader nutritional-quality ideotype that combines enhanced lysine and tryptophan, improved mineral bioavailability, acceptable kernel texture, and stable yield. This approach is consistent with emerging genomic strategies in maize that seek to identify and stack favourable regions for multiple nutritional traits (Jasrotia et al., 2026).

At several loci, one genotype class was represented by only 12–15 lines, resulting in substantial class imbalance that may reduce the precision of the estimated allelic effects and associated P values. Accordingly, the prioritized loci should be regarded as testable hypotheses rather than confirmed causal variants or genes. Fine mapping, local haplotype sequencing, allele-specific expression analysis, and near-isogenic line development will help refine the associated intervals, while functional validation through expression assays, mutant analysis, complementation, or genome editing will be needed to confirm the roles of *Zm00001eb163880*, *Zm00001eb191620*, *Zm00001eb330080*, and other candidates. Because sequencing-derived SNPs may not capture structural variants, rare alleles, copy-number variation, or untagged causal polymorphisms, higher-resolution genotyping or sequencing would further strengthen candidate-gene resolution (Rathore et al., 2026; Arora et al., 2025). Multi-year and multi-location testing will also be required to determine the stability of these loci, together with evaluation of total phosphorus, inorganic phosphorus, mineral concentration, phytate:mineral molar ratios, mineral bioavailability, seed vigor, kernel quality, and grain yield. Such validation will determine whether the favorable alleles and haplotypes identified here can be reliably deployed for QPM biofortification.

## Conclusion

To our knowledge, this study represents the first GWAS-based dissection of natural allelic variation for kernel PA content in maize, specifically in QPM germplasm. The substantial phenotypic variation, highly significant genotypic effects, continuous distribution, and high broad-sense heritability observed in the QPM panel indicate that kernel PA content is a quantitative trait with strong potential for selection. Multi-model GWAS identified five high-confidence marker-trait associations on chromosomes 1, 3, 4, and 7, with chr3:236714544 emerging as the strongest locus based on cross-platform/model support, clear allelic differentiation, and candidate-gene evidence. Integration of positional information, variant-effect prediction, functional annotation, and expression profiling prioritized *Zm00001eb163880*, encoding a plant non-specific lipid-transfer protein/seed storage helical domain-containing protein, as the leading candidate gene at this major locus. In addition, LD-based haplotype analysis identified Hap2/GAC at the chromosome four locus around chr4:176947058 as a favorable haplotype associated with lower PA content. Collectively, these findings suggest that natural variation for PA content in QPM is influenced by seed development, phosphate allocation, signaling, transport, and storage-reserve pathways rather than by canonical PA biosynthesis alone. The identified loci, haplotypes, and prioritized candidate genes provide valuable resources for marker-assisted selection, genomic selection, and precision biofortification of QPM. Validation in independent populations, together with assessment of seed vigour, kernel quality, grain yield, phosphorus fractions, mineral concentration, phytate:mineral molar ratios, and mineral bioavailability, will be essential before breeding deployment.

## Data Availability

The data presented in the study are deposited in the Figshare repository, accession number/DOI: 10.6084/m9.figshare.34022334.
