# Supplementary material for: Multi-model genome-wide association analysis reveals natural allelic variation and candidate loci for kernel phytic acid content in quality protein maize
Source: Front Genet. 2026 Oct 12;17:1925511. doi: 10.3389/fgene.2026.1925511 (PMC13696032; doi:10.3389/fgene.2026.1925511)
Supplement: Supplementary file 1 [file DataSheet1.docx]

**Supplementary Figures**

**Supplementary Figure S1** Distribution of filtered SNP markers across the ten maize chromosomes using a 1-Mb window. The color scale represents the number of SNPs per window.

**Supplementary Figure S2** Genome-wide association signals detected for kernel phytic acid content across AP2021 (E1), PUN2022 (E2), and pooled (E3) datasets. Panels (a), (b), and (c) represent E1, E2, and E3, respectively. The dashed horizontal line represents the LOD = 3.0 significance threshold used for the mrMLM-family analyses.

**Supplementary Figure S3** Representative quantile-quantile (QQ) plot for the GAPIT GWAS analyses of kernel phytic acid content, showing GLM, MLM, FarmCPU, and BLINK models. Observed -log10(P) values are plotted against expected -log10(P) values; the diagonal line represents the expected null distribution.

**Supplementary Figure S4** Bayesian population structure of 249 QPM inbred lines based on filtered SNP markers. (a) Mean log probability of the data [LnP(K)] across tested genetic clusters from K = 1 to K = 10. (b) STRUCTURE ancestry proportion plot at the optimal K = 2, showing the membership coefficient (Q) of each inbred line in the two inferred genetic clusters. (c) Stacked STRUCTURE ancestry plots across tested K values, illustrating changes in inferred ancestry patterns with increasing K. (d) Cluster composition at K = 2, showing the number and percentage of inbred lines assigned to Cluster_1, Cluster_2, and the admixed group. Vertical bars in STRUCTURE plots represent individual inbred lines, and different colours indicate inferred ancestry components.
